# A-to-I RNA editing in the rat brain is age-dependent, region-specific and sensitive to environmental stress across generations

**DOI:** 10.1186/s12864-017-4409-8

**Published:** 2018-01-08

**Authors:** Hiba Zaidan, Gokul Ramaswami, Yaela N. Golumbic, Noa Sher, Assaf Malik, Michal Barak, Dalia Galiani, Nava Dekel, Jin B. Li, Inna Gaisler-Salomon

**Affiliations:** 1 Department of Psychology, University of Haifa, Haifa, Israel; 2 Department of Genetics, Stanford University, Stanford, CA USA; 3 Faculty of Education in Technology and Science, Technion, Haifa, Israel; 4 Faculty of Civil and Environmental Engineering, Technion, Haifa, Israel; 5 Bioinformatics Core Unit, University of Haifa, Haifa, Israel; 6 Department of Marine Biology, Leon H. Charney School of Marine Sciences, University of Haifa, Haifa, Israel; 7 The Mina and Everard Goodman Faculty of Life Sciences, Bar-Ilan University, Ramat-Gan, Israel; 8 Department of Biological Regulation, The Weizmann Institute of Science, Rehovot, Israel; 9 Program in Neurogenetics, Department of Neurology, David Geffen School of Medicine, University of California, Los Angeles, California, Los Angeles USA

**Keywords:** RNA editing, Stress, Transgenerational, Serotonin receptor 2C, Rat, Brain, mmPCR-seq

## Abstract

**Background:**

Adenosine-to-inosine (A-to-I) RNA editing is an epigenetic modification catalyzed by adenosine deaminases acting on RNA (ADARs), and is especially prevalent in the brain. We used the highly accurate microfluidics-based multiplex PCR sequencing (mmPCR-seq) technique to assess the effects of development and environmental stress on A-to-I editing at 146 pre-selected, conserved sites in the rat prefrontal cortex and amygdala. Furthermore, we asked whether changes in editing can be observed in offspring of stress-exposed rats. In parallel, we assessed changes in ADARs expression levels.

**Results:**

In agreement with previous studies, we found editing to be generally higher in adult compared to neonatal rat brain. At birth, editing was generally lower in prefrontal cortex than in amygdala. Stress affected editing at the serotonin receptor 2c (*Htr2c*), and editing at this site was significantly altered in offspring of rats exposed to prereproductive stress across two generations. Stress-induced changes in *Htr2c* editing measured with mmPCR-seq were comparable to changes measured with Sanger and Illumina sequencing. Developmental and stress-induced changes in *Adar* and *Adarb1* mRNA expression were observed but did not correlate with editing changes.

**Conclusions:**

Our findings indicate that mmPCR-seq can accurately detect A-to-I RNA editing in rat brain samples, and confirm previous accounts of a developmental increase in RNA editing rates. Our findings also point to stress in adolescence as an environmental factor that alters RNA editing patterns several generations forward, joining a growing body of literature describing the transgenerational effects of stress.

**Electronic supplementary material:**

The online version of this article (10.1186/s12864-017-4409-8) contains supplementary material, which is available to authorized users.

## Background

Adenosine-to inosine (A-to-I) RNA editing is a modification of double-stranded pre-mRNA catalyzed by adenosine deaminases acting on RNA (ADAR; [1–3]. ADAR enzymes, which include the biologically active ADAR1 and ADAR2, modify a genetically encoded adenosine (A) in double-stranded RNA structures into an inosine (I), read by the cellular machinery as a guanosine (G). A-to-I RNA editing occurs at a multitude of sites on coding as well as non-coding RNA [4, 5], thereby affecting RNA properties and increasing translational diversity. While most editing sites in primates are in untranslated regions (UTRs), many editing sites in coding regions are conserved across species. Several conserved editing sites are located on genes involved in neuronal function [6–8].

Editing levels at individual sites are often developmentally regulated. For example, editing of glutamate and GABA receptors transcripts increases between early development and adulthood [8, 9]. A large-scale analysis of RNA editing changes between embryonic day 15 (E15) and postnatal day 21 (P21) indicates that this increase may occur at many sites and may be uncoupled from changes in ADAR expression [10].

A-to-I RNA editing is particularly prevalent in the brain [11–16], but is not uniform in all brain structures. For example, a high-throughput post-mortem study found higher global editing levels in the cortex compared to cerebellum, but no differences between cortical subregions [17]. In rodents, a high-throughput regional comparison of multiple editing sites is lacking, although examination of editing at individual sites, e.g., *Grik1*, *Gria2* and *Grik2* transcripts, points to baseline and learning-induced differences between hippocampal subregions [9, 18], and variability in other brain structures (e.g., cortex, thalamus and cerebellum [19, 20].

The discovery and characterization of A-to-I RNA editing sites has rapidly advanced in the past decade due to the development of high-throughput, next-generation sequencing methods [21]. Here, we used a recently developed high throughput method that couples multiplex microfluidic PCR and deep sequencing (mmPCR-seq) to measure RNA editing levels with high accuracy, even at sites where low editing levels obscure detection with other deep sequencing techniques [22, 23].

We used the mmPCR-seq method to study editing at 146 pre-selected editing sites in 70 genes in the rat prefrontal cortex (PFC) and amygdala (AMY) at birth and adulthood. Since RNA editing patterns in these regions are altered in stress-related disorders [24–28], we further asked whether exposure to stress in adolescence would affect editing at these sites. The PFC and AMY have different developmental profiles and are differentially affected by stress [29–31].

**Fig. 1:**
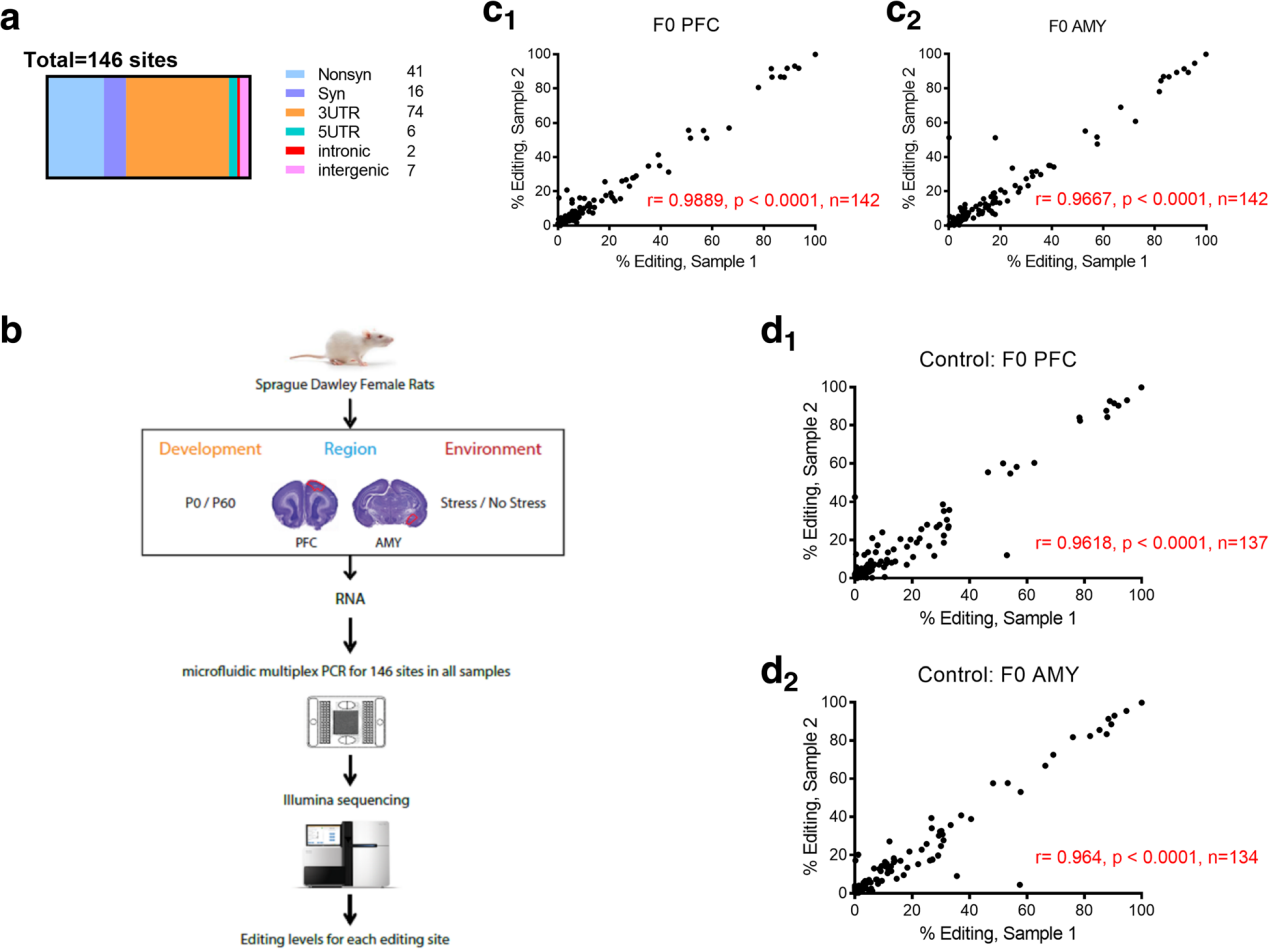
Detection of A-to-I RNA editing in the rat brain with mmPCR-seq. **a** Genomic identity of genes selected for assessment with mmPCR-seq. **b** Schematic diagram of research design and mmPCR-seq. **c** Reproducibility of RNA editing measurements in technical replicates of a control PFC **(c**_**1**_) and AMY (**c**_**2**_) sample. **d** Reproducibility of RNA editing measurements in biological replicates in rat PFC **(d**_**1**_) and AMY (**d**_**2**_)

Our previous studies show that exposure to stress in adolescent female rats prior to reproduction leads to trans-generational effects on behavior, stress-related hormone levels, and cortical gene expression and neuronal morphology [32–36]. Others have shown that some environmentally induced epigenetic states can be inherited [37–39], a process that appears to be mediated by changes to RNA molecules [40, 41]. Here, we asked whether pre-reproductive stress (PRS) in adolescent female rats would impact editing levels at multiple sites in first- and second-generation offspring. We were particularly interested in the serotonin receptor 2c (*Htr2c;* [42, 43]), where editing is affected following early exposure to stress [24, 26, 28, 30, 44–49]. We used the mmPCR-seq method (see Methods and Fig. 1) as well as direct and *Htr2c*-directed next-generation sequencing (NGS; see Methods and Additional file 1: Figure S1) to assess the direct and trans-generational effects of stress on editing at this site.

Our findings point to developmental, regional and stress-induced differences in A-to-I editing in the rat brain, and indicate that the *Htr2c* gene may be particularly vulnerable to the effects of stress across generations.

## Results

### A-to-I RNA editing levels are higher in adult compared to neonatal rat brain

**Fig. 2:**
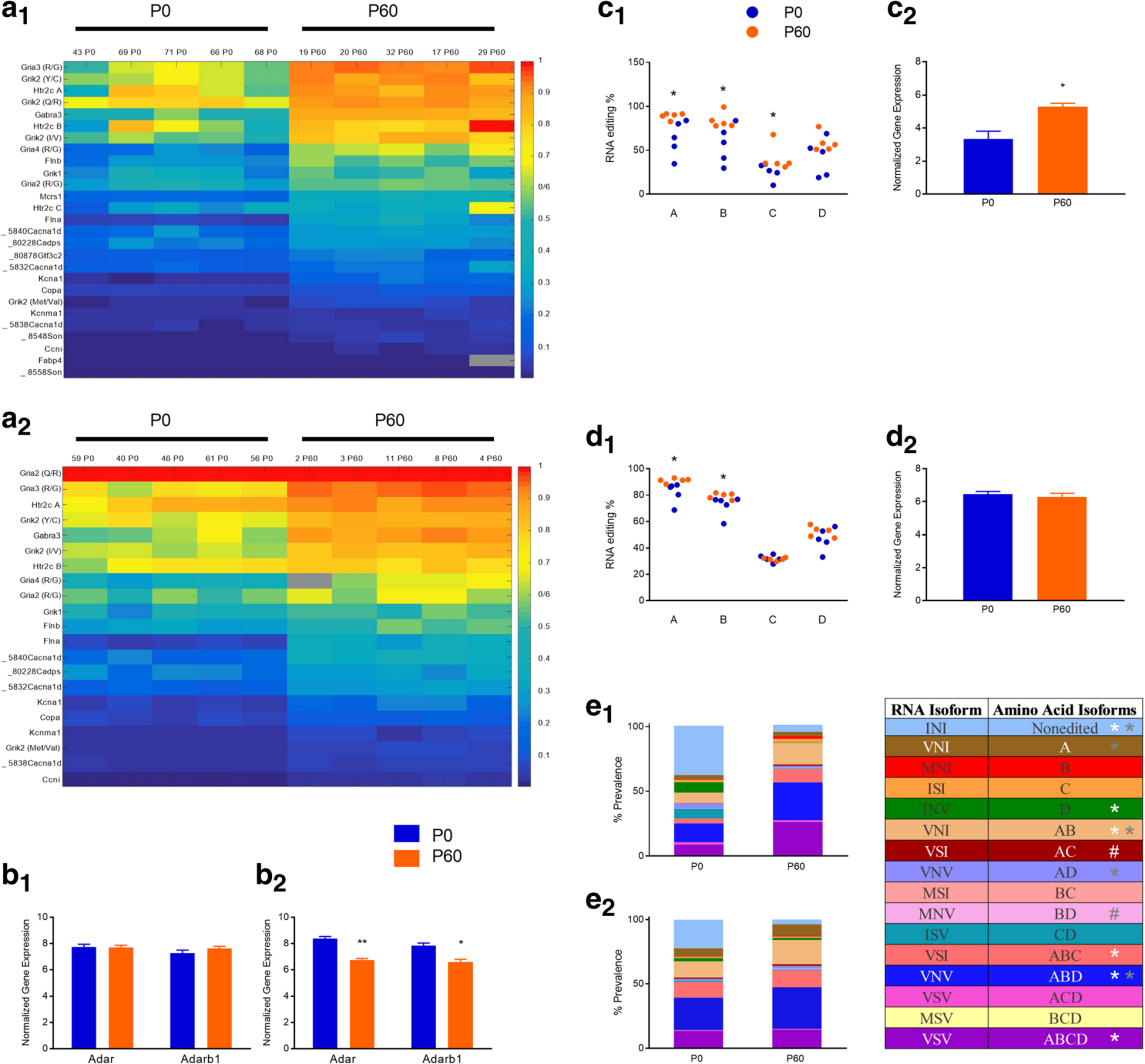
Age-dependent changes in A-to-I RNA editing and gene expression in rat brain. **a** Significant age-dependent changes in RNA editing at non-synonymous editing sites in PFC (**a**_**1**_) and AMY (**a**_**2**_). **b** Age-dependent changes in *Adar* and *Adarb1* mRNA expression in PFC (**b**_**1**_) and AMY (**b**_**2**_). **c** Age-dependent changes in *Htr2c* A-D site editing (**c**_**1**_) and mRNA expression (**c**_**2**_) in PFC. **d** Age-dependent changes in *Htr2c* A-D site editing (**d**_**1**_) and mRNA expression (**d**_**2**_) in AMY. **e** Age-dependent changes in *Htr2c* isoform prevalence in PFC (**e**_**1**_) and AMY (**e**_**2**_). Columns represent individual samples. Rows represent editing sites. *p < 0.05, **p < 0.001, # < 0.075, white, significance in PFC; gray, significance in AMY. **N’s**, RNA editing; P0 5, P60 5; Gene expression (*Adar/b1*), PFC: 12, 5; AMY: 9, 5; *Htr2c*, 11, 5; 5, 9

We asked whether A-to-I RNA editing patterns were different in the PFC and AMY of neonatal (postnatal day (P) 0) vs. adult (P60) rats. In the PFC, we detected editing at 140 sites. Editing levels were significantly different in adults compared to neonate PFC in 50/140 sites; in 49/50 sites, editing in adult PFC was higher than in neonate PFC. In the AMY, we detected editing at 141 sites. Editing levels were significantly different in adults compared to neonate AMY in 70/141 sites; in 67/70 sites, editing in adult AMY was higher than in neonate AMY (Mann-Whitney U test with a Benjamini–Hochberg multiple testing correction, False Discovery Rate (FDR) =0.1; full data in Additional file 2: Table S3**,** SI). The number of sites (categorized by genomic identity) where age-dependent significant changes were found are presented in Table 1**.** As can be seen in Fig. 2a, editing levels at most non-synonymous editing sites increased between P0 and P60 in both PFC (**2a**_**1**_) and AMY **(2a**_**2**_). The mRNA expression of both *Adar* and *Adarb1* in the PFC did not differ between groups (all *p*’s > 0.1, **2b**_**1**_), while in the AMY the expression of both decreased with age (2-Way ANOVA, *Adar*, *F1,12* = 42.508, *p* = 0.00002; *Adarb1*, *F1,12 =* 16.34, *p* = 0.0016; **2b**_**2**_).

**Table 1:** Number of sites where significant changes were observed between neonatal and adult PFC and AMY

	PFC	AMY
Nonsyn	(27/41)	(21/41)
Syn	(4/16)	(6/16)
3UTR	(17/69)	(34/69)
5UTR	(2/6)	(4/6)
intronic	(0/1)	(0/2)
intergenic	(0/7)	(5/7)

At the *Htr2c*, editing was detected at 4 out of the 5 known editing sites (A, B, C and D). We found that in PFC editing at 3 sites (A, B and C) was higher at P60 (**2c**_**1**_). *Htr2c* mRNA expression was also higher in adults compared with neonates (One-Way ANOVA, *F1,14* = 7.357, *p* = 0.0168; **2c**_**2**_). In the AMY, editing at the A and B sites was higher at P60 (**2d**_**1**_), but there were no differences in *Htr2c* transcript levels (**2d**_**2**_).

Each of the five adenosines within the *Htr2c* sequence that encodes amino acids 156–160 can be edited, leading to altered encoding of triplet codons and hence different isoforms of the G-protein-coupled receptor [42, 43]. Figure 2e presents developmental changes in the distribution of *Htr2c* isoforms in PFC and AMY, excluding isoforms containing the E site where editing was not detected. As can be seen, the percentage of the INI (“unedited”) *Htr2c* isoform decreased significantly with age in both PFC (**2e**_**1**_) and AMY (**2e**_**2**_). Interestingly, age-dependent changes in isoform prevalence were generally greater in PFC than in the AMY (Additional file 2: Table S4, SI).

### Different levels of A-to-I RNA editing in PFC vs. AMY at birth but not in adulthood

**Fig. 3:**
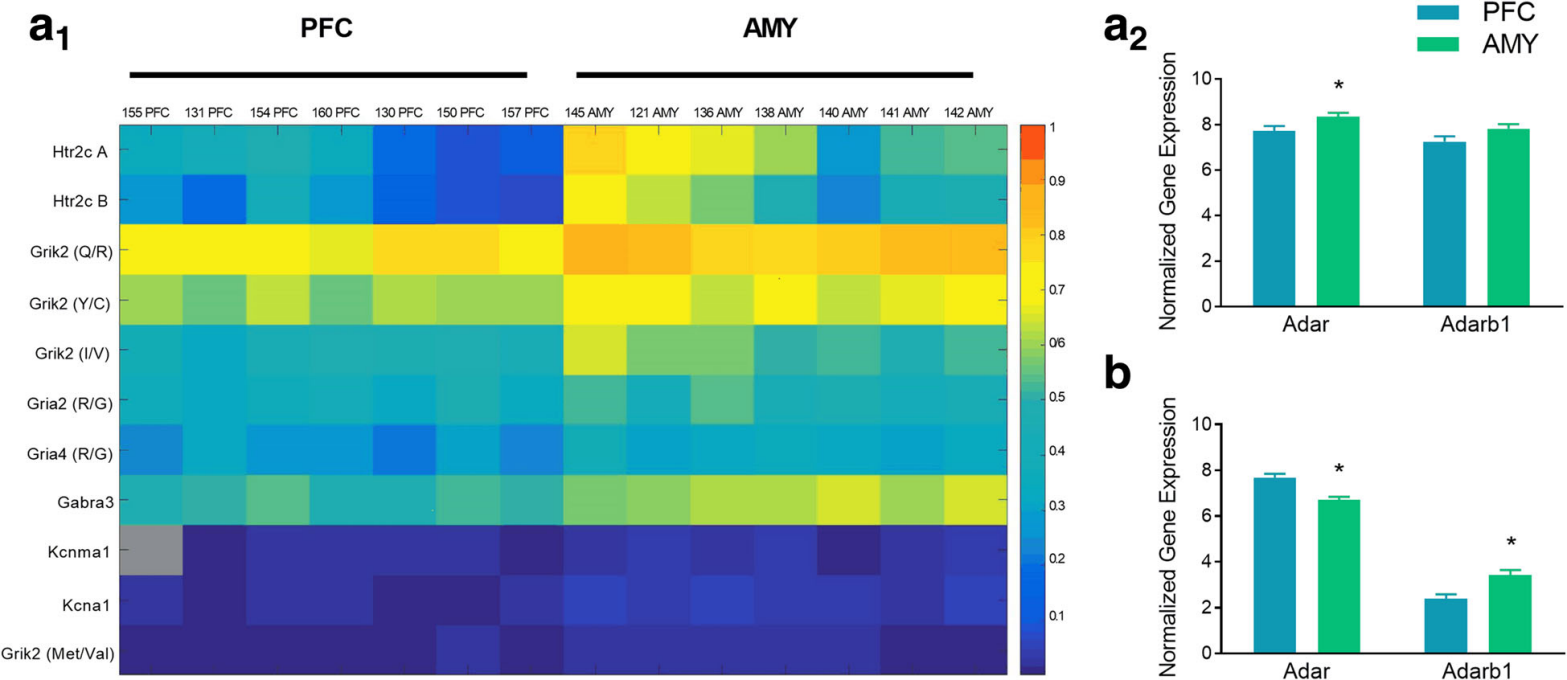
Differences in A-to-I RNA editing and ADAR mRNA expression between PFC and AMY. **a** Significant changes at non-synonymous editing sites (**a**_**1**_) and regional differences in *Adar* and *Adarb1* mRNA expression (**a**_**2**_) levels at P0). **b** regional differences in *Adar* and *Adarb1* mRNA expression at P60. Columns represent individual samples. Rows represent editing sites. *p < 0.05. **N’s**, RNA editing; P0: PFC 7, AMY 7; P60: 5, 5; Gene expression, P0: 12, 9; P60: 5, 5

Since PFC and AMY have different maturation profiles, we asked whether these regions differ in their A-to-I RNA editing patterns in neonatal and adult rats. At P0, we detected editing at all 146 sites. Editing levels at 23 editing sites were different in PFC compared to AMY; in 22/23 sites, editing was lower in PFC (Mann-Whitney U test, FDR = 0.1; see heatmap of non-synonymous site changes in Fig. 3a_1_). At P60, we detected editing at 134/146 sites; no differences between PFC and AMY were detected at any of these sites (see Additional file 2: Table S5 in SI for full PFC and AMY data). The mRNA expression of *Adar* at P0 was lower in PFC than in AMY (2-Way ANOVA, *Adar*, *F1,19* = 4.976, *p* = 0.0379; **3a**_**2**_); there were no differences in *Adarb1* mRNA levels. At P60, mRNA expression of *Adar* was higher in PFC (2-Way ANOVA, *F1,18* = 22.136, *p* = 0.00153), while expression of *Adarb1* was higher in AMY (*F1,18* = 13.826, *p* = 0.0058; **3b**). We examined individual RNA editing and gene expression values for each sample, and asked whether they were correlated. Notably, some samples were analyzed for editing levels or gene expression, but not both (see Methods). As can be seen in Additional file 2: Table S6, there was generally no correlation between editing levels and ADARs or *Htr2c* expression levels, with the exception of the *Gabra3* site where editing levels positively correlated with *Adar* normalized expression.

*Htr2c* A and B editing was lower in PFC (Fig. 3a_1_). Examination of *Htr2c* isoform distribution revealed differences between PFC and AMY in prevalence of 8 isoforms at P0, but only 2 isoforms at P60 (see Additional file 2: Table S7 in SI). A one-way ANOVA revealed lower *Htr2c* mRNA expression in PFC vs. AMY at P0 (*F*_*1,18*_ = 33.068, *p* = 0.00001; Additional file 3: Figure S2A in SI), and at P60 (*F*_*1,18*_ = 9.8, *p* = 0.0140; Additional file 3: Figure S2B in SI).

### *Adar* and *Adarb1* expression levels are differentially affected by stress in PFC and AMY, but A-to-I editing patterns are largely unchanged

**Fig. 4:**
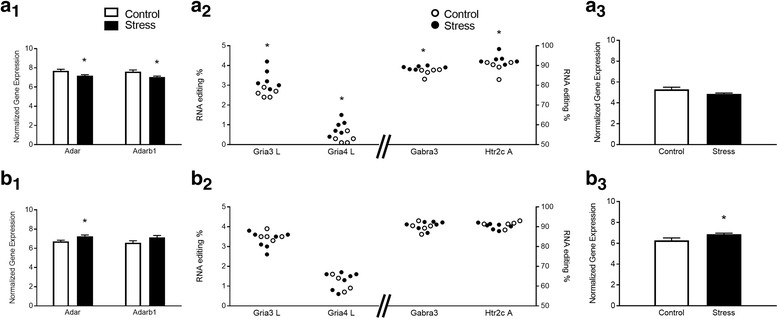
Stress-induced changes in A-to-I RNA editing and related gene expression levels in rat brain. **a** stress-induced changes in *Adar* and *Adarb1* mRNA expression (**a**_**1**_), RNA editing (**a**_**2**_) and *Htr2c* mRNA expression (**a**_**3**_) in PFC. **b** stress-induced changes in *Adar* and *Adarb1* mRNA expression (**b**_**1**_), RNA editing (**b**_**2**_) and *Htr2c* mRNA expression (**b**_**3**_) in AMY. Only significant editing changes are shown. *p < 0.05. **N’s**, PFC: C 5, Stress 6; AMY: 5, 7

**Fig. 5:**
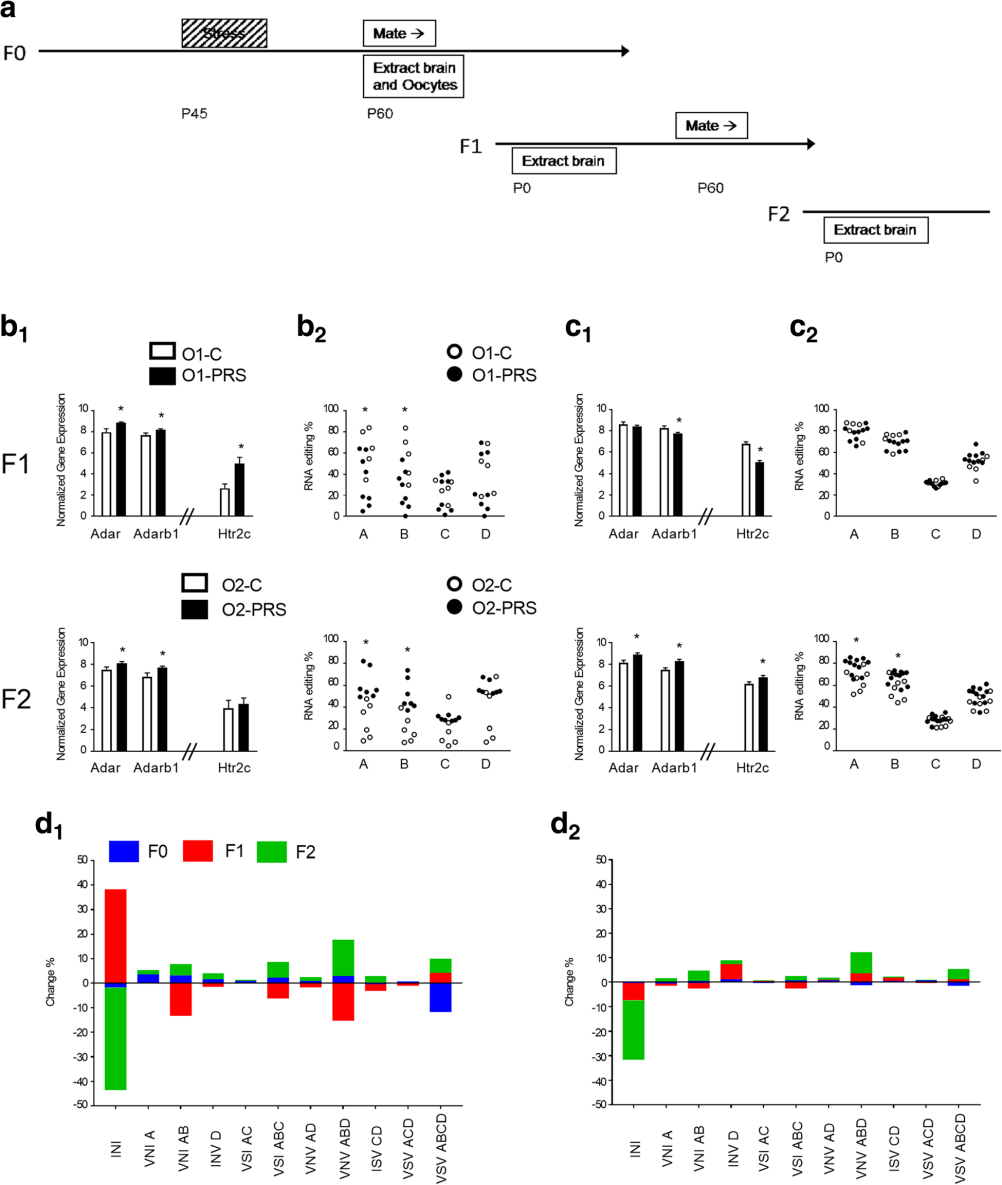
Transgenerational effects of stress on A-to-I RNA editing and related gene expression levels in the rat brain. **a** Experimental Design: Transgenerational transmission of stress effects. **b** mRNA gene expression of *Adar*, *Adarb1* and *Htr2c* (**b**_**1**_), and *Htr2c* A-D site editing (**b**_**2**_) In PFC of F1 (top row) and F2 (bottom row) offspring. **c** mRNA gene expression of *Adar*, *Adarb1* and *Htr2c* (**c**_**1**_), and RNA editing levels of 4 *Htr2c* sites (**c**_**2**_), In AMY of F1 (top row) and F2 (bottom row) offspring. **d** *Htr2c* isoform distribution changes between C and PRS (F0), O1-C and O1-PRS (F1) and O2-C and O2-PRS (F2) in PFC (**d**_**1**_) and AMY (**d**_**2**_). *p < 0.05. **N’s**, PFC: F1, O1-C 6, O1-PRS 8; F2, O2-C 6, O2-PRS 9; AMY: F1, 4, 9; F2, 5, 7

We asked whether an environmental manipulation, i.e., chronic mild stress in adolescent female rats [32–36], would alter RNA editing levels and mRNA expression. We observed a decrease in *Adar* and *Adarb1* mRNA expression 2 weeks after stress (*Adar*, *F1,9* = 7.321, *p* = 0.0241; *Adarb1*, *F1,9* = 0.0160, *p* < 0.05; Fig. 4a_1_). Mann-Whitney analysis with a FDR correction of multiple sites involved in neurotransmission and implicated in the stress response (29 sites in total; [50]; see genes and complete references in Additional file 2: Table S8, SI), revealed significant stress-induced changes in editing at sites coding for serotonin, glutamate and GABA receptors in PFC (**4a**_**2**_; Mann-Whitney U test, *p’s <* 0.05; see Additional file 2: Table S8, **F0** in SI for full results). At the 4 detected editing sites on the *Htr2c* gene, we found that stress led to higher editing levels at the A site in PFC (*p* = 0.027); no changes in *Htr2c* mRNA expression were found (*p* > 0.1; **4a**_**3**_**).** Examination of *Htr2c* isoforms revealed a stress-induced increase in the prevalence of a single isoform (INV) in PFC (ANOVA, *F1,8* = 6.941, *p* = 0.0299; Fig. 5d_1_) and an increase approaching significance in VNI_A_ prevalence was also found in the PFC (*F1,8* = 4.976, *p =* 0.0562).

In the AMY, stress led to an increase in *Adar* mRNA (*F1,10*) = 7.776, *p* = 0.0191; Fig. 4b_1_), but no changes in editing at any of the sites detected with mmPCR-seq (all *p*’s > 0.1). Applying the same analysis to the limited 29-gene set revealed no stress-induced changes (Additional file 2: Table S8). While there were no stress-induced changes in editing at the *Htr2c* (p’s > 0.1; **4b**_**2**_), mRNA expression of *Htr2c* increased (*F1,9* = 6.072, *p* = 0.0334; **4b**_**3**_).

### Pre-reproductive stress (PRS) affects RNA editing and ADAR mRNA expression in first- and second-generation offspring PFC and AMY

Since stress affects editing levels [44] and PRS impacts on behavior in first- and second-generation offspring (O1-PRS and O2-PRS, respectively; [33–36], we asked whether PRS-induced A-to-I editing changes could also be observed across generations (see Fig. 5a for experimental design). As can be seen in Additional file 2: Table S8 in the SI**,** more stress-dependent changes were found in F1 and F2, compared to individuals directly exposed to stress (F0), and the largest number of stress-dependent changes was found in O2-PRS (Mann-Whitney U test, *p’s* < 0.05).

We next analyzed stress-induced changes in *Adar* and *Adarb1* mRNA expression across generations. In PFC**,** PRS to dams increased *Adar* and *Adarb1* mRNA expression of RNA editing enzymes in PFC of F1 (*Adar*, *F1,12* = 8.525, *p* = 0.0128; *Adarb1 F1,12* = 6.174, *p* = 0.0287) and F2 (*Adar*, *F1,13* = 4.983, *p* = 0.0438; *Adarb1*, *F1,13* = 6.015, *p* = 0.0290**)** offspring (**5b**_**1**_**)**.

Focusing on the *Htr2c,* we found increased mRNA expression in F1 (*F1,11* = 8.703, *p* = 0.0132), but not F2, offspring (**5b**_**1**_**)**. Editing at sites A and B decreased in F1 but increased in F2 (*p’s* < 0.05); In F2, increased editing at sites C and D approached significance (*p’s* < 0.075; **5b**_**2**_). Notably, inter-animal variability in editing levels was relatively high compared to variability presented in Fig. 2 and Fig. 4, raising the concern that PFC samples were contaminated during extraction with choroid plexus (CP) tissue (where *Htr2c* expression is high but A and B site editing levels are low) [51]. We thus assessed the expression of the CP marker Coagulation factor V F5 [52] in O1-C (*n* = 5) and O1-PRS (*n* = 6) samples with both low and high A and B site editing. We found high Ct (>32) and dCt (>11.4) values for all samples, indicating that the expression of this marker was low. We found no correlations between A or B site editing and F5 expression (Pearson’s *r* = −0.485 and *r* = −0.453, respectively, NS) nor differences in F5 expression between O1-C (11.93 ± 0.189) and O1-PRS (12.257 ± 0.30) samples (t-test, *p =* 0.4).

In the AMY (Fig. 5c), PRS to dams had no effect on *Adar* but decreased *Adarb1* mRNA expression in F1 offspring (*F1,11* = 5.78, *p* = 0.0349). In F2 offspring, PRS to dams increased *Adar* (*F1,10* = 8.481, *p* = 0.0155) and *Adarb1* (*F1,10* = 6.066, *p* = 0.0335) expression (**5c**_**1**_). At the *Htr2c*, mRNA expression decreased in F1 but increased in F2 offspring of PRS dams (F1, *F1,10 =* 6.224, *p* = 0.0317, F2, *F1,10* = 5.057, *p* = 0.0408; **5c**_**1**_). *Htr2c* editing was unaffected by maternal PRS in F1 offspring; in F2 offspring, editing at sites A and B increased (*p’s* < 0.05; **5c**_**2**_).

As can be seen in Fig. 5d and described above, stress altered the levels of only 1 isoform in the PFC. However, in O1-PRS, the prevalence of 3 isoforms in the PFC (**5d**_**1**_; INI, *F1,10* = 8.055, *p* = 0.0176; VNV_ABD_ *F1,10* = 11.27, *p* = 0.0072; VNI_AB_ *F1,10* = 6.309, *p* = 0.0308; MSI, *F1,10* = 4.172, *p* = 0.068;) and 3 in the AMY (**5d**_**2**_; INV, *F1,10* = 5.35, *p* = 0.0432; ISV, *F1,10* = 5.824, *p* = 0.0364; VNV_AD_ *F1,10* = 5.072, *p* = 0.0480) was significantly different or approached significance compared to F1 offspring of control females (O1-C).

In F2 offspring, the prevalence of 4 isoforms in the PFC (VSV_ABCD_, *F1,10* = 4.969, *p* = 0.0499; INI, *F1,10* = 16.12, *p* = 0.0024; VNV_ABD_ F*1,10* = 13.698, *p = 0.0041*; VSI_ABC_ *F1,10* = 7.659, *p* = 0.0198) and 6 isoforms in the AMY (VSV_ABCD_, *F1,11* = 8.419, *p* = 0.0144; INI *F1,11* = 11.174, *p* = 0.0065; INV, *F1,11* = 8.008, *p* = 0.0163; VNV_ABD_ *F1,11* = 12.352, *p =* 0.0048; VNV_AD_ *F1,11* = 6.053, *p* = 0.0316; VNI_AB_ *F1,11* = 5.696, *p* = 0.0360; VNI_A_ *F1,11* = 3.948, *p* = 0.072), was significantly different or approached significance in O2-PRS compared to F2 offspring of control (O2-C) samples. Notably, the prevalence of the non-edited isoform INI decreased in both PFC and AMY in F2, whereas the prevalence of the most highly edited isoform detected, VSV (edited at sites A, B, C and D) increased in both regions.

To probe for a mechanism for transgenerational changes in RNA editing, we asked whether changes in editing are present in germline cells of stress- exposed female rats. The same 146 editing sites were investigated; in oocytes, editing was detected at 44 sites (Additional file 2: Table S9 in SI). No stress-dependent changes were detected at any of the 16 sites that passed our 3-sample criterion (see Methods). When control and stress-exposed samples were pooled together, significant changes were found between editing levels in brain regions (PFC and AMY) and oocyte samples taken from adult female rats (Additional file 4: Figure S3 in SI). No *Adar* or *Adarb1* mRNA expression was detected in oocytes (not shown).

Since our findings point to a lack of correlation between ADAR expression and editing levels, we asked whether alternative splicing of *Adarb1* is affected by stress. Alternative splicing occurs as a consequence of self-editing of *Adarb1* pre-mRNA and results in a protein that lacks the double-stranded RNA-binding motifs and catalytic deaminase domain. We analyzed whole brain samples from first-generation offspring of control and PRS female rats, and observed an increase in mRNA expression of *Adar* (*F1,18* = 10.181, *p* < =0.0050*)* as well as *Adarb1* (*F1,11* = 5.773, *p* = 0.0287; 6a), similarly to the increase we observed in F1 offspring in PFC (Fig. 5b_1_). We then examined splicing at the +47 alternative splicing site, and found a 14% significant increase in the “inactive” isoform (*F1,11* = 25.493, *p* = 0.0001; 6b).

### Comparison of 3 sequencing methods reveals similar stress-related changes in A-to-I RNA editing levels at the *Htr2c* across generations

We compared mmPCR-seq editing findings at the *Htr2c* to those obtained with Sanger direct sequencing and *Htr2c*-directed NGS. A separate cohort of rats was run in the transgenerational PRS protocol (Fig. 5a), and samples were extracted and analyzed using Sanger direct sequencing; a subset of these samples was also analyzed using *Htr2c*-directed NGS (see Methods and SI for full details). Examining editing levels at 5 sites on the *Htr2c* gene in the AMY of adult control female rats, we found that the three methods showed similar editing levels at each detectable site (the E site was not detected by mmPCR-seq; Additional file 5: Figure S4A, means and SE in Table 2). A non-parametric independent samples median test across groups revealed no technique-dependent differences between editing levels detected at each site.

**Table 2:** Means and SE of editing at *Htr2c* sites detected with three different methods

Method/site	A	B	C	D	E
Sanger (n = 6)	93.01 ± 0.63	82.84 ± 1.38	34.98 ± 1.07	58.58 ± 1.78	6.04 ± 0.42
*Htr2c* -directed NGS (*n* = 3)	87.59 ± 0.57	78.39 ± 1.64	29.76 ± 2.7	54.6 ± 0.97	4.13 ± 0.71
mmPCR-seq (n = 5)	91.2 ± 0.76	79.42 ± 1.06	31.54 ± 0.54	52.3 ± 1.83	ND

ND = not detected

The three techniques also revealed similar effects of stress on offspring: as described above (Fig. 5b_2_), the mmPCR-seq method revealed a decrease in editing in F1 offspring at sites A and B. Using Sanger direct sequencing to evaluate the effect of PRS on F1 offspring brain at birth, we found a similar decrease at sites A and B (Mann-Whitney U test*, p* < 0.05; (Additional file 5: Figure S4B_1_). Similarly, the *Htr2c*-directed NGS method revealed a decrease at sites A and B (Mann-Whitney U test*, p* < 0.05; Additional file 5: S4B_2_).

The distribution of *Htr2c* isoforms in control AMY using NGS or mmPCR-seq is presented in Additional file 5: Figure S4C. As can be seen, NGS detected some rare *Htr2c* isoforms (<1.2% expression) that were not detected with mmPCR-seq. Both techniques revealed that the most prevalent isoforms were VNV (ABD), VNI (AB), VSV (ABCD) and VSI (ABC). Thus, the three methods are comparable and yield similar results.

## Discussion

We used the mmPCR-seq method to detect A-to-I editing at 146 pre-selected sites in two regions in the rat brain. While changes in A-to-I RNA editing in the rat were previously demonstrated in cortical cultures and brain samples [50, 51, 53, 54], this is the first use of mmPCR-seq to quantify editing changes in rat brain tissue at multiple sites. For some sites (e.g., *Celf4*, *Ankrd28*), editing is demonstrated for the first time in the rat; editing levels at these sites is largely conserved with the corresponding human and mouse orthologs. Furthermore, this is the first direct comparison between mmPCR-seq and other sequencing techniques in rat (Illumina-based sequencing of the *Htr2c* and Sanger sequencing), further validating the accuracy of the mmPCR-seq technique as a high-throughput platform. Furthermore, we showed that mmPCR-seq can be used accurately across technical and biological replicates, with little inter-run and inter- animal variability in both brain regions we had examined (Fig. 1).

Examining the neonatal (P0) and adult (P60) rat brain, we found that editing levels were generally higher in adulthood, in both PFC and AMY; the biggest changes in editing occurred in genes related to glutamate or GABA receptors (Additional file 2: Table S3). These data are consistent with previous studies, which examined A-to-I conversions from embryonic development through late adolescence [10, 55]. Since numerous genes undergo robust changes in expression during brain development [56, 57], changes in RNA-related processes (e.g., miRNA expression and RNA editing) along the developmental timeline could reflect the changing demands of the developing brain for post-transcriptional regulation [58]. Interestingly, ADAR enzymes are involved in regulation of aging processes [59], and alterations in A-to-I editing in the brain were observed in aging and in Alzheimer’s disease patient populations [60–62]. The investigation of temporal expression patterns in neural tissue and their regulation across the lifespan can elucidate molecular mechanisms involved in the formation, mature function and degeneration of the brain.

Previous studies examined RNA editing of individual genes in distinct brain regions [9, 18–20], but a direct comparison between PFC and AMY across multiple editing sites has not been performed. The PFC generally reaches structural and functional maturity in late adolescence, typically later than the AMY and other subcortical regions [63, 64]. Here, we found lower A-to-I editing levels in PFC compared to AMY in the neonatal brain; by adulthood, no regional differences were observed. This finding, combined with our observation of a developmental editing increase, implies that RNA editing plays a role in brain maturation and that high editing levels may differentiate mature brain areas from regions that are in the process of neural development.

**Fig. 6:**
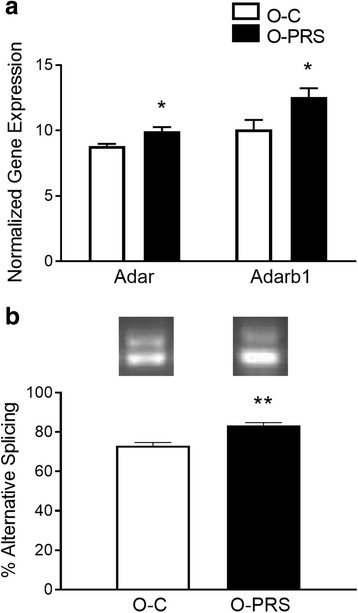
Relationship between mRNA expression and alternative splicing of *Adarb1* in rat brain. **a** mRNA expression of *Adar* and *Adarb1* in O-C and O-PRS brains. **b** Reverse Transcription-PCR of alternative splicing in *Adarb1*. Upper and lower bands represent inclusion or exclusion, respectively, of the 47 nt sequence in O-C and O-PRS brain at P0 (top). Mean *Adarb1* alternative splicing levels in O-C and O-PRS brain at P0. *p < 0.05, **p < 0.001. **N’s**, *Adar*: O-C 10, O-PRS 9; *Adarb1*: 10, 10; Splicing: 9, 9

Changes in *Adar* and *Adarb1* expression levels did not correlate with changes in editing levels. For example, *Adar*-mediated editing at sites A and B of the *Htr2c* gene increased between birth and adulthood, whereas *Adar* transcript levels remained unchanged (Fig. 2). Conversely, environmental stress induced an increase in *Adar* mRNA, but no change in *Htr2c* A site editing (Fig. 4). Several previous studies also found no correlation between editing activity and ADAR mRNA or protein expression levels, pointing to complex mechanisms of ADAR-mediated editing regulation and substrate-to-enzyme specificity [10, 65–68]. For example, *Adarb1* dimerization patterns [69] and recently discovered interactions between ADARs and miRNA/siRNA pathways [70, 71] can contribute to the non-linear relationship between editing rates and editing enzyme expression levels. In support of this, we found here that an increase in *Adarb1* mRNA expression in fact may reflect elevated levels of the “inactive”, self-edited isoform (Fig. 6).

Serotonergic signaling plays an important role in neurodevelopment [72]. *Htr2c* editing was previously detected in both PFC and AMY [25, 46, 73, 74], and editing at its 5 sites is affected by environmental stress [26, 28, 30, 44–46]. Using the mmPCR-seq method, we were able to detect editing at A, B, C and D sites, but not at the E site, on the *Htr2c* gene. Editing at the E site is observed less frequently than the other 4 sites on this gene in humans and rodents [61, 75, 76]. The absence of E site editing in the present study could be due to regional differences or to detection issues; notably, a previous study using the mmPCR-seq technique also found no editing at this site [61].

Similarly to previous studies [10, 77–79], we found higher editing levels at the A and B sites in adult PFC and AMY (site C editing was also elevated in PFC). An analysis of age-dependent changes in transcript isoform prevalence revealed a decrease in the unedited isoform (INI), and an increase prevalence of the partly edited VSV and VNV isoforms (Fig. 2), which are the two most prevalent isoforms in rat/mouse and human brain [6, 76]. Editing of the G protein-coupled *Htr2c* transcript affects constitutive receptor signaling, cell surface expression and G protein coupling efficiency [6, 42, 80]. Thus, an increase in the prevalence of ‘edited’ isoforms in the adult brain may signify altered regulation of several second messengers such as inositol trisphosphate and cAMP, leading to alterations in the dynamics of Ca^2+^ signaling [75].

Closer examination of our findings reveals high variability in A and B site editing levels in neonatal (e.g., Fig. 5b_2_) compared to adult PFC (e.g., Fig. 2c_1_). This could be due to the greater variability in cell populations (e.g., neurons vs. astrocytes, or pyramidal cells vs. interneurons) in the PFC at this early developmental stage. Since RNA editing levels can vary as a result of cell type composition [81] and since the *Htr2c* plays an important role in neurodevelopment [72], future studies should examine A and B site editing in distinct cell populations during development.

In our hands, chronic mild stress did not lead to dramatic changes in editing at most sites. A hypothesis-based analysis of editing changes in stress- and learning-related genes revealed alterations in 4 editing sites, including 2 sites where editing led to no change in amino acid sequence (‘synonymous’; *Gria3*, *Gria4*) and the *Gabra3* and *Htr2c* A sites, “non-synonymous” editing sites where editing results in amino acid replacement. Editing in *Gabra3* mRNA, coding for the alpha-3 subunit of the GABA-A receptor, leads to an isoleucine to methionine (I/M) change in the third transmembrane region. The editing event affects receptor trafficking and gating kinetics, and is developmentally regulated [8, 82–84], Additional file 2: Table S3 in present findings). Here we shown that stress affects *Gabra3* editing, presumably affecting GABA-A receptor function. Stress-induced editing changes at the *Htr2c* are consistent with previous studies [28, 44–46] and are associated with stress-related disorders [24–26, 28, 30, 45, 85]. Alterations in ADAR expression levels in PFC and AMY, as we observed following stress, were also observed in previous rodent studies and in psychopathological populations, although conflicting findings have been reported [24–28]. Notably, stress in our study was administered during adolescence, a period of particular vulnerability to negative environmental intervention [86].

We and others have shown that the effects of stress on behavior and HPA axis functionality can be transmitted across several generations [32–36, 40, 87–89]. Several studies point to opposite effects in F1 and F2 or to effects that emerge fully or are magnified in second- or third-generation offspring [16, 89–91]. Here, we found that editing levels differed between offspring of stress-exposed and control female rats at many sites in both PFC and AMY, and this difference was especially pronounced in F2.

Stress affected *Htr2c* A and B site editing in all three generations; editing decreased in F1 but increased in F2, where the most pronounced changes were observed. An examination of isoform prevalence in offspring revealed a decrease in the ‘unedited’ isoform (INI) and an increase in VNV in F1 offspring of stress-exposed rats, previously shown to exhibit an anxiogenic phenotype [36]. Decreased INI prevalence was observed in anxiety-prone strains of mice [25].

While the impact of dam-pup interactions or gestational changes cannot be ruled out, they are unlikely to account for the effects of stress on editing in subsequent generations, since stress in F0 occurred prior to reproduction and the F2 generation was generated through the paternal germline. Other studies implicate epigenetic mechanisms, particularly changes in small non-coding RNA, in the long-term, multigenerational transmission of adverse environmental interventions [16, 40, 92, 93]. Interestingly, RNA editing enzymes interact with RNA-binding proteins that control access of specific microRNAs to their mRNA targets, which may provide a mechanism for transgenerational transmission [94–96]. Such mechanisms should be carefully investigated in future studies, since they may provide new insight on mechanisms of inherited phenotypic variation and disease risk. Future studies should also examine sex differences, which were not possible to determine in the present investigation due to the small sample size.

## Conclusions

We used mmPCR-seq to accurately detect A-to-I RNA editing in the rat brain. Several novel editing sites were found, and we showed that editing in the rat brain is region-specific and sensitive to developmental processes. The footprints of stress on editing, particularly at the serotonin receptor *Htr2c*, can be detected across several generations. While it is widely accepted that environmental factors can induce epigenetic alterations, the detection of stress-induced editing changes in offspring of affected individuals can contribute to our understanding of causal factors in variability of behavioral phenotypes in psychiatric health and disease.

## Methods

### Animals

Adolescent female Sprague-Dawley rats and adult males used for mating were purchased from Envigo (Jerusalem). Housing conditions (except during the stress procedure) included wood-flake bedding, ad lib food and water, 12 h artificial lighting during the day (07-19 h), and temperature maintained at 22 ± 2oC.

Animals were randomly distributed across groups (see Experimental procedure below). The number of animals per group appears in the Figures. Rats were handled in accordance with the NIH guidelines for the Care and Use of Laboratory Animals, 8th edition [97]. The study was approved by the University of Haifa Committee on animal experimentation (294/13, 351/14, 197/10).

### Experimental procedure

#### Developmental/regional differences experiment

Male and female neonatal (P0) and female adult (P61–66) rats were sacrificed, and their PFC and AMY were extracted for mmPCR-seq and gene expression analysis (see below).

#### Stress experiment

Adolescent (P40–45) female rats were group housed (4 or 6 rats per cage) in 56x35x19cm cages. Cages were randomly divided into control (C) and PRS groups. PRS rats underwent a 7-day unpredictable stress procedure, as previously described [32–36]. Randomly-selected females from C and PRS groups were sacrificed two weeks after the end of the stress procedure (P61–66), and PFC and AMY were extracted for mmPCR-seq and gene expression analysis.

#### Transgenerational stress experiment

C and PRS females from the Stress experiment were mated with behaviorally-naïve males. Oocytes from unmated C and PRS rats were removed following hormonal treatment, as previously described [36]. F1 generation: O1-C and O1-PRS were sacrificed at P0 and their PFC and AMY were extracted for RNA editing and gene expression analysis. Pups were raised undisturbed until P30, then weaned and raised in same-sex, same-condition groups of 4–6. Adult male O1-C and O1-PRS rats were mated with naïve females. F2 generation: O2-C males and O2-PRS males were sacrificed at P0. PFC and AMY were extracted for gene expression and RNA editing analysis. To compare the effects of chronic mild stress on editing across generations, we examined PFC, AMY and oocytes from C and PRS rats, as well as PFC and AMY from F1 and F2 offspring.

#### Methodology comparison experiment

A separate cohort of adolescent female rats was subjected to the stress protocol described above. AMY was removed 24 h following the stress procedure for Sanger direct sequencing and NGS (see below). Remaining females were mated, and whole brain was removed from F1 progeny of O-C and O-PRS pups at P0, and analyzed using Sanger direct sequencing and NGS. Data from male and female offspring were pooled together in all experiments.

### Breeding

Breeding was conducted as previously described [36]. Briefly, a behaviorally naïve male rat was introduced into a cage with 2 female rats and was removed 7 days later. Female rats were returned to their home cage; pregnancy was verified by weekly weighing. Each pregnant rat was moved to a 37x30x19cm cage 7 days prior to parturition.

### Brain removal and dissection

Rats were sacrificed by decapitation and brains were removed and placed on dry ice. Brains were mounted on a cryostat and bilateral samples from PFC and AMY (see Fig. 1) from adult and neonatal rats were removed according to coordinates specified by the Rat Brain Atlas [98] or the Atlas of the Neonatal Rat Brain [99] using 1.0 or 0.5 mm punches, respectively. All samples were immediately placed on dry ice and kept at -80 °C until further processing.

### RNA extraction and cDNA preparation

RNA from oocytes and brain was extracted as previously described [35, 36]. RNA quantities were determined using a Nanodrop 2000 spectrophotometer (Thermo Scientific, Wilmington, DE) or Qubit fluorometer (Thermo Fisher Scientific, Waltham, MA). The 260:280 nm absorbance ratio was measured to assess RNA quality; samples were excluded if the ratio was outside the range of 1.7–2.0, or if RNA concentration was too low. RNA quality was verified in 12 randomly-selected samples using the Bioanalyzer TapeStation 2200 (Agilent Technologies Inc., Santa Clara, CA, USA); The RNA integrity Number (RIN) of all samples was >8. PureLink®RNA Mini Kit (Ambion) was used to further purify some of these samples. cDNA was prepared using iScript™ Advanced cDNA Synthesis Kit (Bio-Rad, Hercules, CA, USA) or High Capacity cDNA Reverse Transcription kit (Applied Biosystems, Foster City, CA or Quanta Bioscience, Manchester, UK). cDNA used in the mmPCR-seq experiment were purified with Agencourt® AMPure® XP beads (Beckman Coulter, Brea, CA, USA) and concentrated using SpeedVac.

### Editing site selection for mmPCR-seq

Since there is no rat-specific data on the RADAR database (v.2; http://rnaedit.com/), which stands as a comprehensive collection of rigorously annotated RNA editing sites [100], we selected editing sites by comparing sites that are conserved between mouse and rat, lifting over all of the known mouse editing sites onto the rat genome (rn4, Nov. 2004, Baylor HGSC v. 3.4). Using this method, 964 conserved editing sites were identified. We excluded 757 sites that are fully intergenic or intronic. Finally, we compared our selected sites against published rat RNA-seq [101] and the UCSC browser annotations in silico, and filtered low coverage sites (<1%), as well as sites where primer design was challenging due to gene size. Of the 177 editing sites we tested, we detected editing at 146 sites (Fig. 1a; (see Additional file 2: Table S1 in SI for the list of sites where editing was detected)).

### Primer preparation and mmPCR-seq

We quantified A-to-I RNA editing using a technique that combines mmPCR with deep sequencing, as previously described [22, 23]; see Fig. 1b). Briefly, we designed 48 pools of 2–3 plex multiplex PCR primers (see Additional file 2: Table S2) to amplify 146 sites; some primer sets amplified more than a single editing locus. The sizes of the amplicons ranged from 150 to 350 bp. We loaded cDNA and primer pools into the 48.48 Access Array IFC (Fluidigm) and performed target amplification as previously described [22, 23]. PCR products of each sample were then subjected to a 15-cycle barcode PCR and pooled together. All pools were combined at equal volumes and purified via Agencourt® AMPure® XP beads. The library was sequenced using NextSeq 500 (Illumina, USA) with 76 bp paired-end reads.

Paired-end reads were combined and mapped onto the genome (rn4) using BWA samse allowing 9 mismatches per read [102]. We aligned the sequencing reads to a combination of the reference genome and 70 bp exonic sequences surrounding known splicing junctions from available gene models (obtained from the UCSC genome browser). We quantified editing levels by dividing the fraction of reads containing a ‘G’ nucleotide by the total reads at each editing site. Only sites covered by ⩾50 mmPCR-seq reads were included. We analyzed two technical replicates for some of the samples to verify concordance between runs; as can be seen in Fig. 1c, there was a highly significant, positive correlation between editing levels in technical replicates taken from the same PFC or AMY sample. The correlation between biological replicates (2 control PFC or AMY samples) was also high and significant (Fig. 1d). For each comparison, we excluded editing sites that had less than 3 biological replicates, and samples where >30% of editing sites were missing (except in the oocytes data). The raw sequencing data is deposited in the Sequence Read Archive (GEO accession number GSE99214). Custom scripts used to process data are available upon request.

### Cluster analysis of *Htr2c* isoforms from mmPCR-seq data

A-to-I RNA editing of the *Htr2c* gene (*Rattus norvegicus* 5-hydroxytryptamine serotonin receptor 2C) occurs at 5 sites (A through E) and can result in 32 mRNA variants that translate to 24 protein isoforms. We performed editing cluster analysis as previously described [61]. Briefly, we aligned the reads with samtools mpileup (v0.1.18; s [102]), to get the sequence information per genomic location, keeping the data of the original reads. Using an in-house computer program, we were able to find the editing sites in the *Htr2c* cluster in each read. We used only reads that included all cluster editing sites. For each sample we summed the different combinations of actual editing locations, and found the percentage from the total number of reads that covered all the locations for each isoform. Isoforms that include the E site were not included in the calculations, since editing was not detected at this site.

### Library preparation for sanger direct sequencing

The PCR amplifications were performed in a volume of 25 μL containing 10 μL ReddyMix (Thermo Scientific, UK), 1 μL forward primer (10 μM) 5′AGATATTTGTGCCCCGTCTG3′, 1 μL reverse primer (10 μM) 5′CTGAAACTCCTATTGATATTGCCC3′, 6 μL ultrapure H2O and 2 μL cDNA from each sample. Amplification was carried out in a thermocycler T professional Basic Gradient (Biometra) with the following program: 5 min at 94 °C, followed of 40 cycles of amplification of 30 s at 94 °C, 30 s at 56 °C and 30 s at 72 °C, and a final extension step of 5 min at 72 °C. The PCR products were then purified with the Wizard SV Gel and PCR Clean-Up System (Promega, Madison, WI, US) and purified products were sent to Hy Laboratories (Rehovot, Israel) for direct PCR sequencing using the reverse primer. Automated sequencing was carried out using BigDye terminator v1.1 Cycle Sequencing Kit (Applied Biosystems, Inc.). The percentage of edited mRNA molecules in a pool of specific *Htr2c* mRNAs was determined as previously described [60, 103] by calculating the peak area of the edited nucleotide (G) versus the sum of G and A peak areas at each editing site using ABI PRISM 3730 DNA analyzer v. 5.2 (Applied Biosystems, Foster City, CA).

### *Htr2c*-directed NGS

Pilot studies revealed that a 1-step PCR process, where the forward primer consisted of the adaptor sequence followed by a full complementary Read1 sequence, a barcode and a gene-specific sequence, resulted in an inefficient reaction and product that was low in yield and quality, possibly as a result of primer self-dimerization.

A randomly-selected subset of samples sequenced using the Sanger direct sequencing method were also sequenced using a 2-step *Htr2c*-directed NGS technique, which we adapted from [50] and modified according to techniques described by [104, 105] in order to optimize the efficiency of the reaction and the yield and quality of the product (see Additional file 1: Figure S1 for description of Illumina-based NGS), as detailed below.

### Primer design for *Htr2c-*NGS

Two sets of primers were designed for a two-step PCR reaction (Additional file 2: Table S2). Primers were designed to flank the 125-bp sequence containing the 5 editing sites in the *Htr2c* gene, and therefore detect all possible isoforms resulting from editing at the *Htr2c* gene.

The forward primer of the first PCR step (F1) included a 20 bp sequence that partially complements the Illumina Read1 Sequencing primers, followed by a 6 nt sample-specific barcode and a 20 bp gene-specific sequence. The complementary 26 bp reverse primer (R1) was comprised of a gene-specific sequence designed to span exon 5 and 6, and detect the spliced variant RNA2 but not the inactive alternative spliced RNA3 variant.

The second PCR step was designed in order to attach the Illumina adaptor sequences that bind to the Illumina flow cell. The forward primer (F2) is a TruSeq Universal Adapter, as used by [50], and consisted of the 20 bp adaptor TruSeqv3 flow cell binding sequence followed by the 5 bp linker (a key between the adaptor and the forward primer), and the full 33 bp Read1 complementary sequence. The reverse Primer (R2) included the 22 bp reverse flow cell adaptor followed by a 12 bp universal primer [50] and the 26 bp reverse primer R1.

### *Htr2c* library preparation and NGS

For Step 1, cDNA from 14 samples was amplified using the F1 and R1 primers with Phusion High-Fidelity DNA Polymerase (New England Bio Labs) in 25 μl reactions (0.25 μl Phusion DNA polymerase, 5 μl 5× Phusion HF buffer, 2 μl cDNA, 200 μM dNTPs (Applied Biosystems, Foster City, CA) and 500 nM of each primer). The PCR amplification protocol was 98 °C for 30 s, followed by 35 cycles of 98 °C for 10 s, 63 °C for 30 s, and 72 °C for 30 s and a final extension step of 72 °C for 2 min. Amplicons were purified with Agencourt AMPure XP beads (pilot studies revealed that the optimal product: beads ratio was 1:1.8) and a final elution was performed with 20 μl water. 5 μl of the purified product was run on a 1% agarose gel to ensure the presence of the desired 151 bp band. Sample concentrations and quality were analyzed by nanodrop or Qubit fluorometer. Twenty-five ng from 6 or 8 samples were pooled together. For Step2 PCR, the pooled cDNA amplicons (150 or 200 ng) were added to 4 μl Phusion DNA polymerase, 20 μl 5× Phusion HF buffer, 200 μM dNTPs and 500 nM of each F2 and R2 primer, in a 100 μL reaction. Amplification was performed using an identical PCR amplification protocol to the Step1 program, and was run for only 8 cycles. Amplicons were purified again with Agencourt AMPure XP beads, with a product: beads ratio of 1:1 with a final elution of 40 μl ddH2O. Five μl of the purified product were run on a 1% agarose gel to ensure the presence of a single 223 bp band. Samples were pooled together with equimolar concentrations (100 ng from each 8-sample pool and 75 ng from the 6-sample pool) and subjected to 50 bp single end sequencing with the Illumina HiSeq2000 (Illumina, San Diego, CA, USA) at the Technion Genome Center.

### Analysis of *Htr2c*-NGS data

Samples barcoded and pooled for sequencing were demultiplexed using FASTX-Toolkit (http://hannonlab.cshl.edu/fastx_toolkit/). To reduce the proportion of base-call errors at editing sites, only reads having quality scores >20 (phred scale) at >80% of the sites were kept.

Fastq reads were aligned and mapped to the 32 variant reference sequences using Bowtie2/TopHat2 [106, 107]. The counts of read-sequences mapped to each variant, and the properties of each alignment, were retrieved using scripts based on Pysam module (http://code.google.com/p/pysam/). Another method of mapping that counted and estimated proportions of A/G/T/C nucleotides (nucleotide pileup) at each edited site separately was used. Fastq reads were mapped using REDItoolDenovo (REDItools toolkit; [108]), against a single reference sequence in which the 5 edited sites of *Htr2c* contained N nucleotide, and allowing <=6 mismatches in the alignment. The two independent methods had high similarity which indicates that the results are technically reliable.

### Quantitative real-time PCR

Quantitative real-time PCR (qRT-PCR) was performed as previously described [36]. Some of the samples used for RNA editing assessment (mmPCR-seq or *Htr2c*-NGS) were also assessed for mRNA expression of RNA editing enzymes and *Htr2c*. In some cases, additional samples were added for qRT-PCR analysis, since RNA quantities were insufficient for both RNA editing and gene expression studies. **Primers** (see Additional file 2: Table S2) for all genes except for F5 (see below) were designed using *Primer3* [109] software, and synthesized by Integrated DNA Technologies (Coralville, IA). Primer suitability was determined using standard curve analysis, melting curve analysis, and linearity at threshold [110, 111]. Data analysis is performed on dCt/ relative to the housekeeping gene hypoxanthine phosphoribosyl transferase (HPRT). In figures, data is represented as normalized gene expression (10-dCt). F5 was detected using a FAM-labeled Taqman probe and primer set (Applied Biosystems, CA, USA; assay ID Rn01483178_m1) and glyceraldehyde-3-phosphate dehydrogenase (GAPDH) (assay ID Rn99999916_s1) as an internal control.

### Analysis of *Adarb1* alternative splicing

As a measure of *Adarb1* activity, we assessed the levels of self-editing-dependent alternative splicing at intron 4 of the *Adarb1* transcript [112, 113] as described [18]. RT-PCR was run using intron-spanning primers that detect the 103 bp unedited (spliced) active isoform or the 150 bp edited (alternately spliced, +47 nt) inactive isoform. Amplicons were run on 1% agarose gel and band peaks intensities were measured with imageJ [114]. Percent alternative splicing was calculated by dividing the upper band (+47 nt) intensity of each sample by the total intensity of both bands.

### Statistical analyses

Data were analyzed with SPSS 20 Statistics software (IBM, Chicago, Illinois) and R package version 3.2.5. Heatmaps were made using Matlab (MathWorks, Natick, MA). In the global editing experiment, a nonparametric Mann-Whitney U test with Benjamini–Hochberg multiple testing correction was used, with FDR =0.1 [61]. We used specifically constructed R package scripts (available upon request). For the *Htr2c* experiment and gene expression, splicing data and isoforms distribution analysis of variance (ANOVA), multivariate analysis of variance (MANOVA), LSD for post-hoc, independent samples median test were used. Significance level was set at *p* < .05. Results that approach significance were defined as .05 ≤ *p* ≤ .075. For the specific *Htr2c* sites Mann-Whitney U test was used. The Pearson test was used to estimate covariance between parameters.

## Additional files

Additional file 1: Figure S1. A Flow chart of sample preparation using the Illumina-based *Htr2c*-direcetd NGS. (PDF 92 kb)

Additional file 2: Table S1. RNA editing sites detected with mmPCR-seq. **Table S2**. Primer sequences used for mmPCR-seq, Real-Time PCR and *Htr2c*-directed NGS. **Table S3**. % RNA editing in PFC and AMY of neonatal (P0) vs. adult (P60) rats. **Table S4**. Age-dependent changes in *Htr2c* isoform prevalence in PFC and AMY. **Table S5**. % RNA editing in neonatal (P0) and adult (P60) PFC vs. AMY. **Table S6**. *Htr2c* and ADARs correlations with significant non-synonymous editing sites. **Table S7**. Changes in *Htr2c* isoform prevalence in PFC vs. AMY at P0 and P60. **Table S8**. The effects of PRS on RNA editing at learning- and stress-related genes in F0, F1 and F2. **Table S9**. Statistical analysis of editing differences between oocytes, PFC and AMY. (XLSX 92 kb)

Additional file 3: Figure S2. *Htr2c* mRNA expression in PFC vs. AMY at P0 (**A**) and P60 (**B**). *p < 0.05, **p < 0.001. **N’s**, P0: PFC 11, AMY 9; P60: 5, 5. (TIFF 209 kb)

Additional file 4: Figure S3. A-to-I RNA editing in oocytes, AMY and PFC of adult female rats. Editing sites where % editing are high are presented in the top part of the figure; sites where % editing are low (0–4%) are presented in the bottom part. **N’s**, PFC, 11, AMY 12, Oocytes 5–12. (TIFF 584 kb)

Additional file 5: Figure S4. Comparison of different methods assessing A-to-I editing levels at the *Htr2c*. (**A**) Editing levels at the 5 sites on the *Htr2c* gene detected with mmPCR, *Htr2c*-directed NGS and Sanger direct sequencing. (**B**) Changes in *Htr2c* RNA editing detected in F1 offspring of control (O-C) and stress-exposed (O-PRS) neonatal rat brain, detected using the Sanger direct sequencing method (**B**_**1**_) and *Htr2c*-directed NGS (**B**_**2**_). (**C**) Comparison of *Htr2c*-directed NGS and mmPCR-seq to detect the distribution of *Htr2c* isoforms in the AMY of control adult female rats. * *p* < 0.05. **N’s**, (**A**): Sanger 6, NGS 3, mmPCR-seq 5; Sanger: O-C 17, O-PRS 18; NGS: 5, 6. (TIFF 558 kb)
