# Supplementary material for: A-to-I RNA editing in the rat brain is age-dependent, region-specific and sensitive to environmental stress across generations
Source: BMC Genomics. 2018 Jan 8;19:28. doi: 10.1186/s12864-017-4409-8 (PMC5759210; doi:10.1186/s12864-017-4409-8)
Supplement: Supplementary file 1 — A Flow chart of sample preparation using the Illumina-based Htr2c-direcetd NGS. (PDF 92 kb) [file 12864_2017_4409_MOESM1_ESM.pdf]

Fig S1

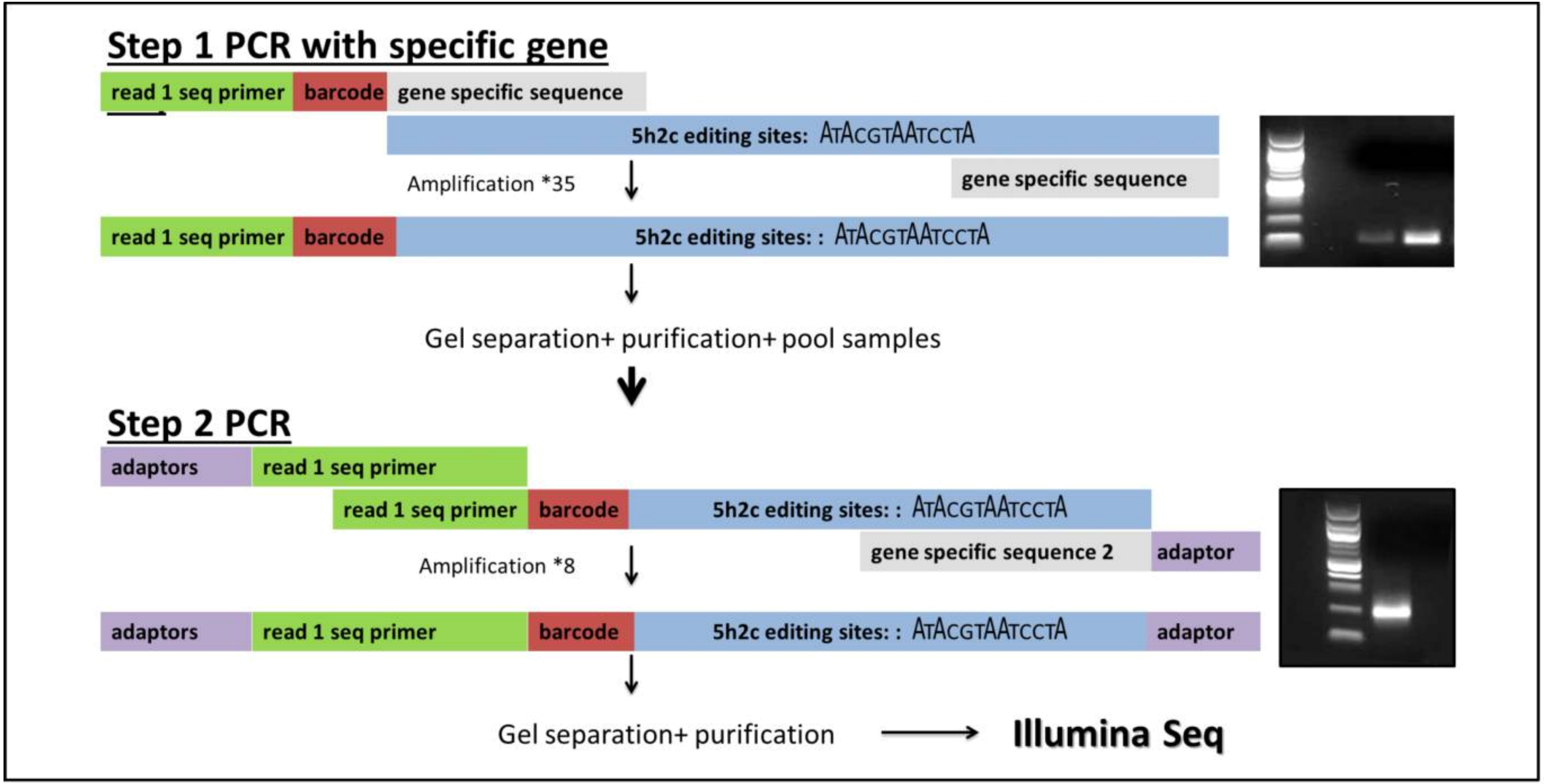
Step 1 PCR with specific gene
read 1 seq primer
barcode
gene specific sequence
5h2c editing sites: ATACGTAATCCTA
Amplification *35
gene specific sequence
read 1 seq primer
barcode
5h2c editing sites: : ATACGTAATCCTA
Gel separation+ purification+ pool samples
Step 2 PCR
adaptors
read 1 seq primer
read 1 seq primer
barcode
5h2c editing sites: : ATACGTAATCCTA
Amplification *8
gene specific sequence 2
adaptor
adaptors
read 1 seq primer
barcode
5h2c editing sites: : ATACGTAATCCTA
adaptor
Gel separation+ purification
Illumina Seq
