# Supplementary figures and images for: A-to-I RNA editing in the rat brain is age-dependent, region-specific and sensitive to environmental stress across generations
Source: BMC Genomics. 2018 Jan 8;19:28. doi: 10.1186/s12864-017-4409-8 (PMC5759210; doi:10.1186/s12864-017-4409-8)

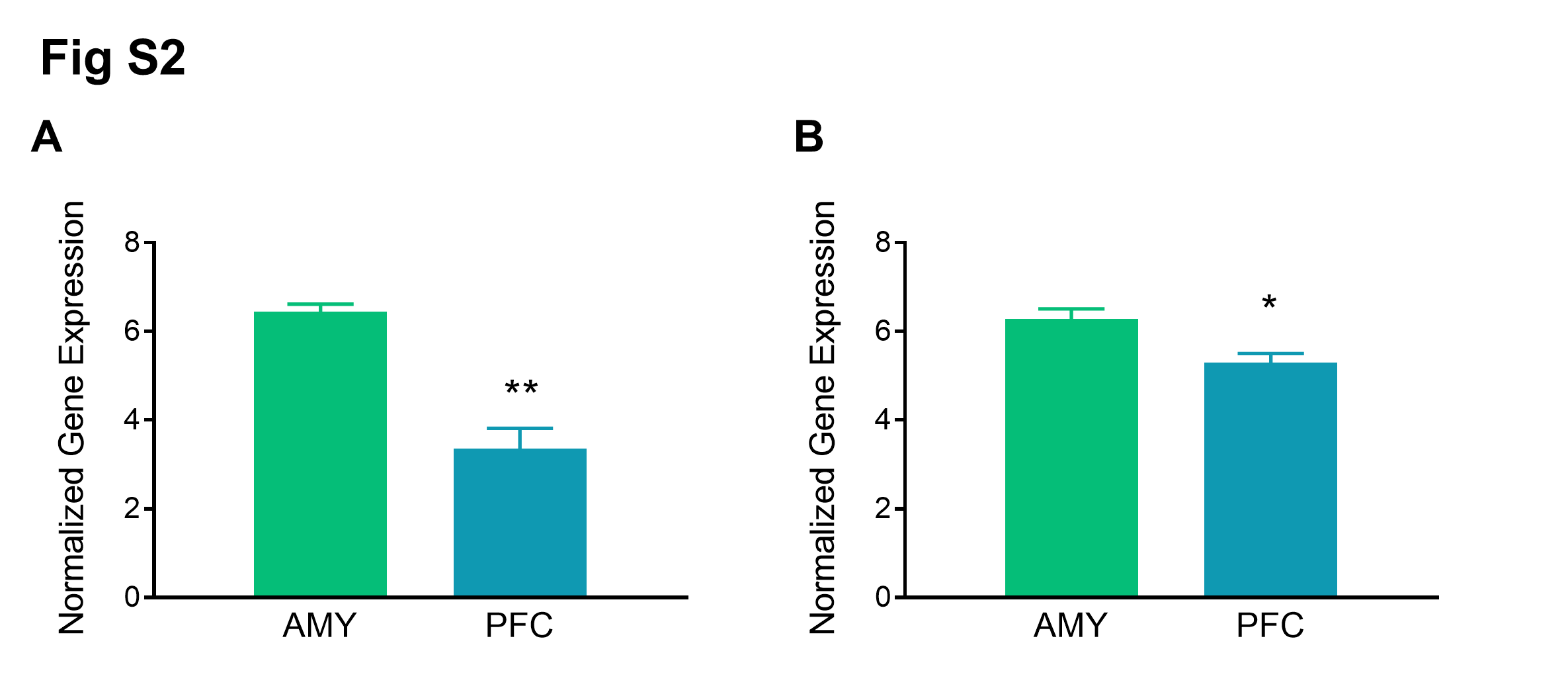

Supplement: Supplementary file 3 — Htr2c mRNA expression in PFC vs. AMY at P0 (A) and P60 (B). *p < 0.05, **p < 0.001. N’s, P0: PFC 11, AMY 9; P60: 5, 5. (TIFF 209 kb) [file 12864_2017_4409_MOESM3_ESM.tif]

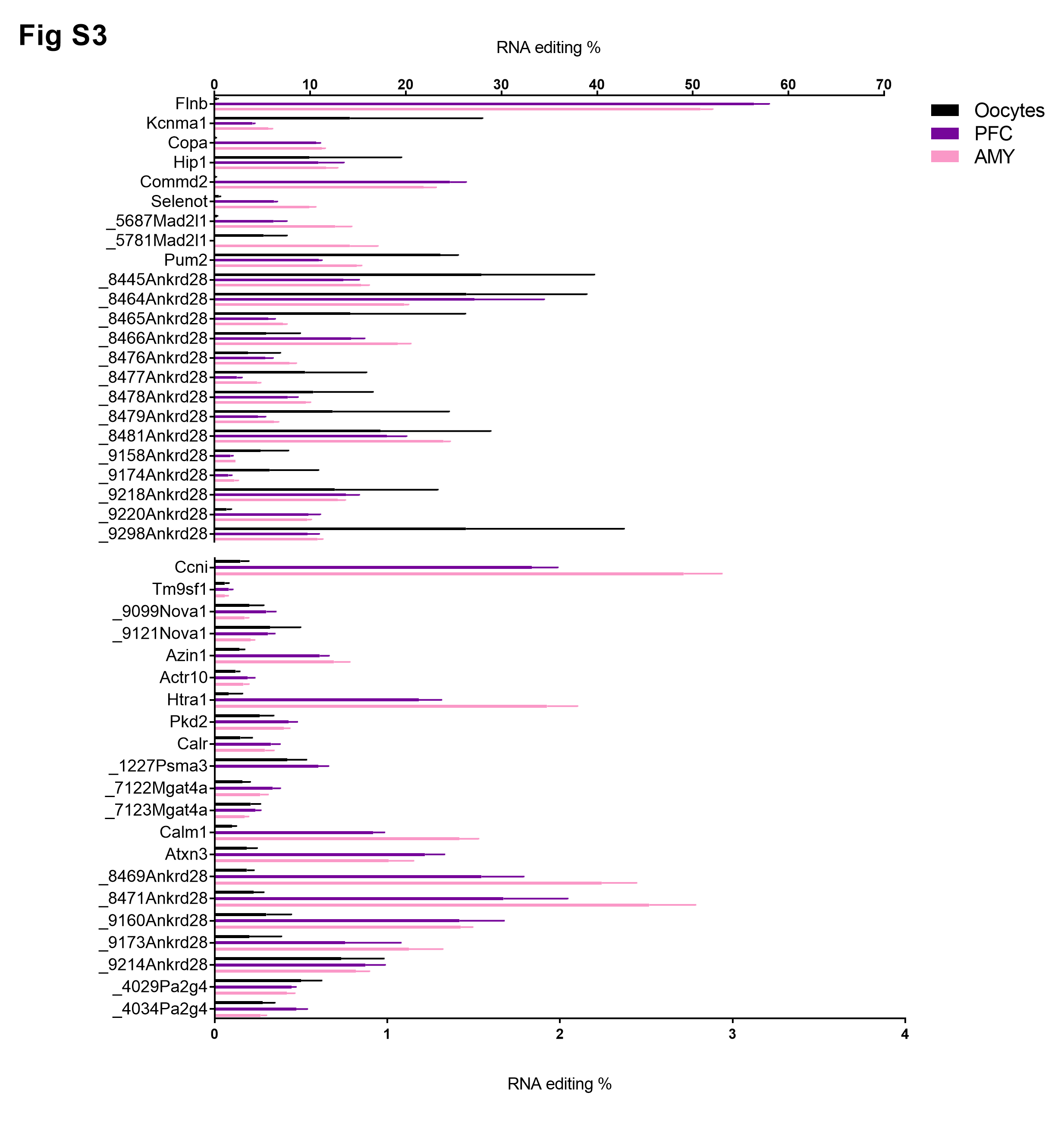

Supplement: Supplementary file 4 — A-to-I RNA editing in oocytes, AMY and PFC of adult female rats. Editing sites where % editing are high are presented in the top part of the figure; sites where % editing are low (0–4%) are presented in the bottom part. N’s, PFC, 11, AMY 12, Oocytes 5–12. (TIFF 584 kb) [file 12864_2017_4409_MOESM4_ESM.tif]

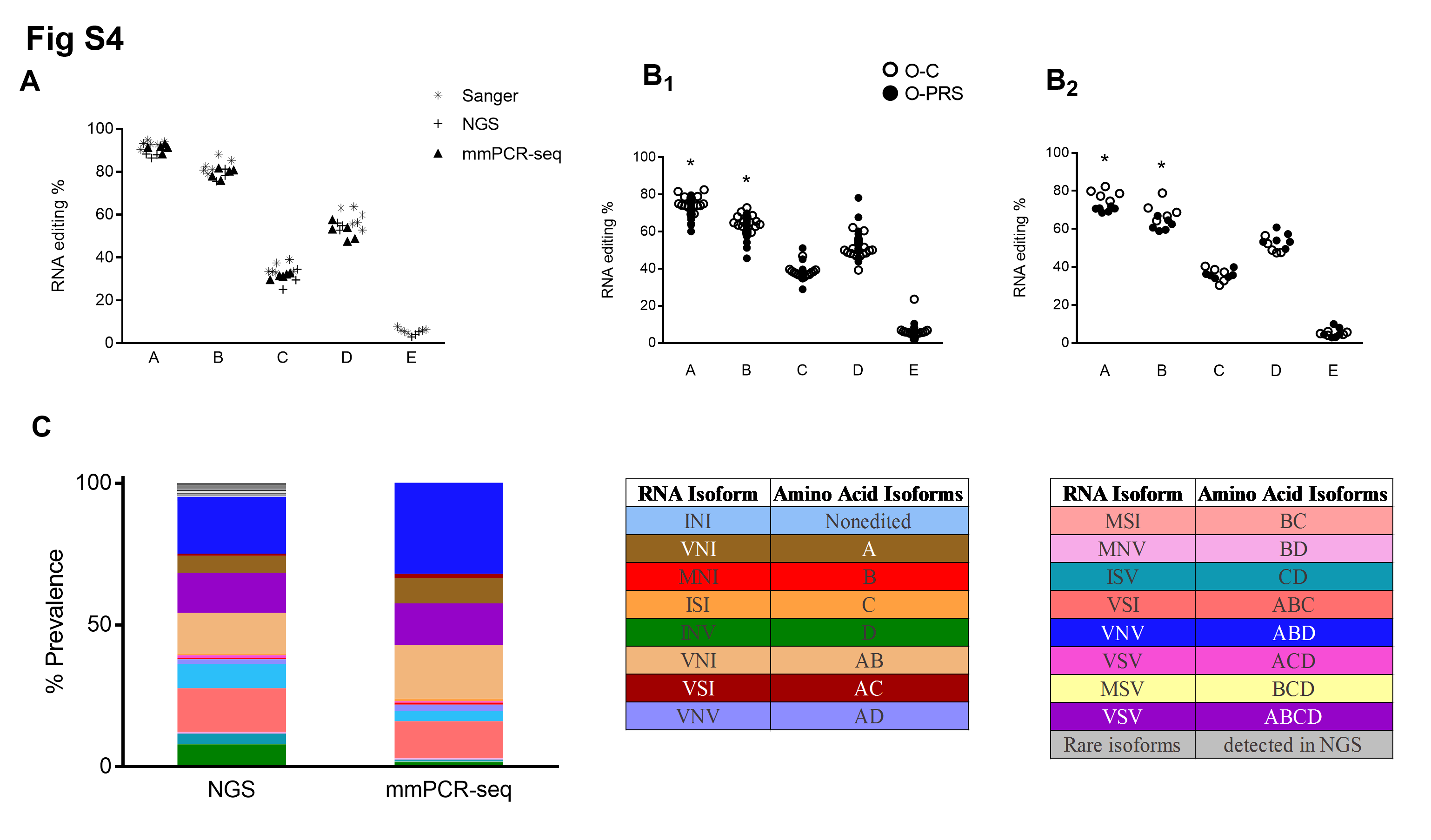

Supplement: Supplementary file 5 — Comparison of different methods assessing A-to-I editing levels at the Htr2c. (A) Editing levels at the 5 sites on the Htr2c gene detected with mmPCR, Htr2c-directed NGS and Sanger direct sequencing. (B) Changes in Htr2c RNA editing detected in F1 offspring of control (O-C) and stress-exposed (O-PRS) neonatal rat brain, detected using the Sanger direct sequencing method (B1) and Htr2c-directed NGS (B2). (C) Comparison of Htr2c-directed NGS and mmPCR-seq to detect the distribution of Htr2c isoforms in the AMY of control adult female rats. * p < 0.05. N’s, (A): Sanger 6, NGS 3, mmPCR-seq 5; Sanger: O-C 17, O-PRS 18; NGS: 5, 6. (TIFF 558 kb) [file 12864_2017_4409_MOESM5_ESM.tif]
